# Functional analysis by minigene assay of putative splicing variants found in Bardet–Biedl syndrome patients

**DOI:** 10.1111/jcmm.13147

**Published:** 2017-05-13

**Authors:** María Álvarez‐Satta, Sheila Castro‐Sánchez, Guillermo Pousada, Diana Valverde

**Affiliations:** ^1^ Grupo de Biomarcadores Moleculares (BB1) Departamento de Bioquímica, Genética e Inmunología Facultad de Biología Universidad de Vigo Vigo Spain; ^2^ Instituto de Investigación Sanitaria Galicia Sur (IISGS) Vigo Spain

**Keywords:** ciliopathies, Bardet–Biedl syndrome, splicing variants, minigene assay, nonsense‐mediated decay, genetic counselling

## Abstract

Bardet–Biedl syndrome (BBS) and Alström syndrome (ALMS) are rare diseases belonging to the group of ciliopathies. Although mutational screening studies of BBS/ALMS cohorts have been extensively reported, little is known about the functional effect of those changes. Thus, splicing variants are estimated to represent 15% of disease‐causing mutations, and there is growing evidence that many exonic changes are really splicing variants misclassified. In this study, we aimed to analyse for the first time several variants in *BBS2*,*ARL6/BBS3*,*BBS4* and *ALMS1* genes predicted to produce aberrant splicing by minigene assay. We found discordance between bioinformatics analysis and experimental data when comparing wild‐type and mutant constructs. Remarkably, we identified nonsense variants presumably resistant to nonsense‐mediated decay, even when a premature termination codon would be introduced in the second amino acid (p.(G2*) mutation in *ARL6/BBS3* gene). As a whole, we report one of the first functional studies of *BBS/ALMS1* variants using minigene assay, trying to elucidate their role in disease. Functional studies of variants identified in BBS and ALMS patients are essential for their proper classification and subsequent genetic counselling and could also be the start point for new therapeutic approaches, currently based only on symptomatic treatment.

## Introduction

Bardet–Biedl syndrome (BBS, MIM #209900) and Alström syndrome (ALMS, MIM #203800) are pleiotropic disorders belonging to the group of ciliopathies, produced by defects in primary cilium structure and/or function (widely reviewed in 1, 2). Both syndromes frequently show overlapping phenotypes that make an accurate diagnosis difficult, especially at early stages 3. Thus, BBS has as primary features retinal dystrophy, obesity, polydactyly, renal and urogenital anomalies and cognitive impairment 4. On the other hand, ALMS is characterized by retinal dystrophy, obesity, sensorineural hearing loss, type 2 diabetes mellitus and congenital cardiomyopathy 5.

Regarding genetic background, 21 *BBS* genes (*BBS1‐C8orf37/BBS21*) have been involved in BBS to date 6, 7, 8, whose mutations would explain around 80% of affected patients, whereas *ALMS1* is the only gene involved in ALMS until now 9. Extensive interfamilial and intrafamilial variability in clinical presentation have been reported for both syndromes, which in the case of BBS cannot be fully explained by genetic heterogeneity when members of the same family harbour the same mutation(s), suggesting other mechanisms involved such as epigenetic regulation. In addition, BBS is commonly inherited as an autosomal recessive disease, but an oligogenic model of inheritance has been proposed for some families, whereby mutant alleles in *BBS* genes and other alleles in different loci with a modifier role would interact to modulate the penetrance and expressivity of the disease 10, 11. However, ALMS is considered a classical autosomal recessive syndrome. The high genetic heterogeneity associated with BBS also contributes to delay the molecular diagnosis, although the progressive introduction of powerful sequencing tools in clinical practice is helping to detect the disease‐causing mutations in a fast and easier way 12, 13. Moreover, mixed BBS/ALMS phenotypes have been widely described 14, 15, which further complicates the molecular analysis and subsequent genetic counselling.

Although mutational screening studies of BBS/ALMS cohorts have been extensively reported (compiled in 7, 16), little is known about the functional effect of all those variants, which are largely increasing due to massive sequencing projects involving genetic disorders 12, 13, 17. Many of these missense, synonymous or intronic changes are indeed variants of unknown significance (VUS), being necessary to elucidate their biological role and impact on phenotype, especially when no other causal changes are identified, the classification as polymorphism is questionable, or a modifier effect is suspected.

Thus, it is well accepted that synonymous changes can affect the conformation and stability of mRNA molecules, the splicing process, the accuracy of translation and the three‐dimensional structure of resulting proteins 18, 19. In addition, there is increasing evidence that exonic mutations (nonsense, missense and also silent) could play an important role in pre‐mRNA splicing *via* creation and/or elimination of exonic splicing enhancer and exonic splicing silencer (ESE/ESS) sequences, unlike their predicted effect only at protein level 18, 20. Thus, it is believed that up to 25% of exonic variants could alter splicing process 20, inducing exon skipping, intron retention, generation of new splice sites or activation of cryptic splice sites 18, 21. This might be especially determining in genes with large exons, such as several *BBS* genes. As a whole, splicing variants are estimated to represent at least 15% of disease‐causing mutations 12, 22. Their biological implications will depend on the balance between type and quantity of mRNA isoforms, and the resulting protein, what can alter the severity or penetrance of disease 12, 23.

Based on this previous background, we aimed to analyse here several variants in *BBS2* (MIM *606151)*, ARL6/BBS3* (MIM *608845)*, BBS4* (MIM *600374) and *ALMS1* (MIM *606844) genes found in clinically diagnosed patients with BBS, which were predicted to alter the normal splicing process. To our knowledge, this is the first study of several variants associated with BBS/ALMS by minigene assay, contributing to the few functional studies reported until now.

## Materials and methods

### Ethics statement

This study adhered to the tenets of the Declaration of Helsinki and was approved by an ethics committee (*Comité Ético de Investigación Clínica de Galicia*‐Galician Ethical Committee for Clinical Research, 2006/08). Informed consent was obtained from all study participants or their guardians.

### Nomenclature and selection of variants

We selected *BBS* and *ALMS1* variants previously identified in our Spanish BBS cohort. The molecular analysis was performed by different approaches, including BBS/AS Asper Ophthalmics genotyping microarray (Asper Biotech, Tartu, Estonia), direct sequencing and/or homozygosity mapping [24, 25, unpublished data].

All variants were numbered according to the recommendations of the Human Genome Variation Society (http://www.hgvs.org/mutnomen/), considering the first nucleotide of the translation initiation codon ATG as c.1 nucleotide. The following cDNA reference sequences were used: *ALMS1*‐NM_015120.4, *BBS2*‐ENST00000245157, *ARL6/BBS3*‐ENST00000463745, *BBS4*‐ENST00000268057 and *MKKS/BBS6*‐ENST00000347364.

### Bioinformatics

Once selected, we carried out an exhaustive *in silico* analysis to predict potential alterations in the donor/acceptor splice sites using the following tools: *NetGene2* (available at http://www.cbs.dtu.dk/services/NetGene2/) 26, *Neural Network SPLICE* (*NNSplice*) 0.9 version from the Berkeley Drosophila Genome Project (http://www.fruitfly.org/seq_tools/splice.html) 27, *Human Splicing Finder* (*HSF*) 2.4.1 version (http://www.umd.be/HSF/) 28 and *Automated Splice Site Exon Definition Server*‐*ASSEDA* (http://splice.uwo.ca/) 29. Finally, ESE sequences were predicted with *Relative Enhancer and Silencer Classification by Unanimous Enrichment‐Rescue ESE* (http://genes.mit.edu/burgelab/rescue-ese/) 30. Default settings were used in all predictions.

We also analysed the effects at protein level for missense variants with *PolyPhen‐2* (http://genetics.bwh.harvard.edu/pph2/) 31, *Pmut* (http://mmb.pcb.ub.es/PMut/) 32 and *SIFT* (http://sift.jcvi.org/) 33.

### Minigene assay

Exonic and at least 150 bp of flanking 5′ and 3′ intronic sequences from genomic DNA of controls or relatives were PCR‐amplified using *Phusion High‐Fidelity DNA Polymerase* (Thermo Scientific, Lithuania), with primers carrying restriction sites for *XhoI* and *NheI* (Table S1). PCR products were subcloned into the splicing reporter pSPL3 vector (kindly provided by Dr JM Millán) using *T4 DNA Ligase concentrated* (New England Biolabs, Ipswich, MA, USA). Minigene constructions were confirmed by direct sequencing using *BigDye* ^*®*^ *Terminator v3.1 Cycle Sequencing Kit* (Applied Biosystems™, Carlsbad, CA, USA), with primers 5′‐CATGCTCCTTGGGATGTTGAT‐3′ (forward) and 5′‐ACTGTGCGTTACAATTTCTGG‐3′ (reverse). Then, we performed site‐directed mutagenesis with *QuikChange II XL Site‐Directed Mutagenesis Kit* (Agilent Technologies, Santa Clara, CA, USA) to obtain the mutant sequence in each case (Table S2).

For minigene assay, 2 × 10^5^ COS‐7 cells (kindly provided by Dr JM Millán) were grown to 80‐90% confluency in 2 ml of Dulbecco's modified Eagle medium, DMEM (Gibco^®^, Grand Island, NY, USA), supplemented with 10% foetal bovine serum (PAA Laboratories, Pasching, Austria), 1% penicillin/streptomycin (Gibco^®^, Grand Island, NY, USA) and 1% L‐glutamine (Lonza, Basel, Switzerland) in six‐well plates (Jet Biofil^®^, Guangzhou, China) at 37°C and 5% CO_2_ atmosphere. Cells were transiently transfected with 2 μg of plasmid (wild‐type or mutant) using *Lipofectamine* ^*®*^ *2000* (Thermo Scientific, Carlsbad, CA, USA), in duplicate. After 36 hrs, RNA was extracted and purified using *Nucleic Acid and Protein Purification NucleoSpin* ^*®*^ *RNA II* (Macherey‐Nagel, Düren, Germany), and then cDNA was synthesized from 1 μg of RNA with GeneAmp *Gold RNA PCR Core Kit* (Applied Biosystems™, Branchburg, NJ, USA). This cDNA was amplified with *Phusion High‐Fidelity DNA Polymerase* (Thermo Scientific, Lithuania), using SD6 (5′‐TCTGAGTCACCTGGACAACC‐3′) and SA2 (5′‐ATCTCAGTGGTATTTGT GAGC‐3′) primers. Finally, PCR products were visualized in 2% agarose gels containing ethidium bromide to study the transcript band pattern and then identify potential changes in splicing process by direct sequencing.

Technical controls of our minigene assay protocol were performed to confirm it was valid in order to properly interpret the results. Tissue samples from patients were not available for further verifications at protein level.

## Results

Firstly, we chose seven variants (Table 1) to further analyse by bioinformatics tools to predict any effect in splicing process: two in *BBS2* (p.(Y89C) and p.(R275*)), one in *ARL6/BBS3* (p.(G2*)), one in *BBS4* (c.77‐6A>G), two in *MKKS/BBS6* (c.986‐29A>T and c.1161+58A>G) and one in *ALMS1* (p.(H3882Y)) gene. As a result, five changes were predicted to alter the recognition of donor/acceptor sites and create/eliminate splice sites and/or ESE sequences, as a score of at least ‘2’ (two positive predictions with splice site prediction tools) was obtained (Table 2). Variants *ALMS 1* p.(H3882Y), with three of five positive predictions, and *BBS2* p.(R275*), with four of five, reached the highest scores. The intronic changes c.986‐29A>T and c.1161+58A>G in *MKKS/BBS6* gene were excluded because only one splice site prediction program (apart from *Rescue ESE*) yielded alterations. More data that could support the pathogenicity of these five changes are described below.

**Table 1:** *BBS/ALMS1* variants selected for performing bioinformatics analysis using splicing prediction tools

Variant	Gene	Exon (bp)	Proband status	Segregation data	Pathogenicity^c^	Other mutations	Allele frequency^g^
c.266A>G/p.(Y89C)	*BBS2*	2 (228)	Heterozygote	Father: carrier (hz)	*Pmut*: Pathological (NN output: 0.8531^d^)	–	0.0005
c.823C>T/p.(R275*)	*BBS2*	8 (136)	Homozygote	Parents: carriers (hz)	NA	–	0.0002
c.4G>T/p.(G2*)	*ARL6/BBS3*	2 (150)	Homozygote	Parents: carriers (hz)	NA	–	0.0000
c.77‐6A>G^a^	*BBS4*	3 (80)	Heterozygote	Mother and affected brother: non‐carriers	NA	p.(Q284*)/p.(Q284*) (*BBS10)*	0.0169
c.986‐29A>T^a^	*MKKS/BBS6*	4 (176)	b	NA	NA	f	0.845
c.1161+58A>G^a^	*MKKS/BBS6*	4 (176)	b	NA	NA	f	0.194
c.11641C>T/p.(H3882Y)	*ALMS1*	17 (121)	Heterozygote	Affected sister: non‐carrier	Benign^e^	p.M390R/p.M390R (*BBS1*)	0.0010

Abbreviations: *bp—*base pairs, *hz*—heterozygote, NA—not applicable. *Proband status*,* other mutations* and *segregation data* information are detailed only when one family was involved. ^a^These variants are located in the following introns: intron 2‐3 (c.77‐6A>G), intron 3‐4 (c.986‐29A>T), intron 4‐5 (c.1161+58A>G). ^b^ *MKKS/BBS6* intronic changes were found in several families of our cohort, in both homozygous and heterozygous states. ^c^Pathogenicity analysis at protein level was performed with three different software tools (*PolyPhen‐2*,* Pmut* and *SIFT*). Only data from pathological outcomes are shown. ^d^NN output bigger than 0.5 is considered pathological. ^e^The three software tools considered this missense change as benign. ^f^Some of the involved families harboured additional mutations. ^g^General allele frequencies were obtained from ExAC database (http://exac.broadinstitute.org/). The following cDNA reference sequences were used: ENST00000245157 (*BBS2*), ENST00000463745 (*ARL6/BBS3*), ENST00000268057 (*BBS4*), ENST00000347364 (*MKKS/BBS6*) and NM_015120.4 (*ALMS1*).

**Table 2:** Bioinformatics analysis of the *BBS/ALMS1* variants previously selected for this study

Variant	Type of splice site	*NetGene2*	*NNSplice*	*HSF*	*ASSEDA*	*Rescue ESE*	Score
c.266A>G/p.(Y89C) *BBS2*	Acceptor	**Two acceptor sites are removed**	Neutral	Neutral	Neutral	Neutral	**2**
	Donor	**Score for donor site decreases from 55 to 51**	The WT consensus sequence is not recognized	**A new donor site is created**	Neutral		
c.823C>T/p.(R275*) *BBS2*	Acceptor	**Score for acceptor site decreases from 97 to 82 and a new site is created**	Neutral	**New acceptor sites are created**	**One potential branch point is broken**	**A new ESE is created**	**4**
	Donor	The WT consensus sequence is not recognized	The WT consensus sequence is not recognized	**New donor sites are created**	**Score for donor site increases from 3.2 to 4.6**		
c.4G>T/p.(G2*) *ARL6/BBS3*	Acceptor	**Score for acceptor site decreases from 16 to 15 and three acceptor sites are removed**	Neutral	**New acceptor sites are created**	Neutral	Neutral	**2**
	Donor	Neutral	Neutral	**New donor sites are created**	Neutral		
c.77‐6A>G *BBS4*	Acceptor	The WT consensus sequence is not recognized	The WT consensus sequence is not recognized	**Two new acceptor sites are created**	**Score for acceptor site decreases from 4.5 to 4.1**	Neutral	**2**
	Donor	Neutral	Neutral	Neutral	Neutral		
c.986‐29A>T *MKKS/BBS6*	Acceptor	The WT consensus sequence is not recognized	Neutral	Neutral	**Score for acceptor site decreases from 2.5 to 1.9**	**Two ESEs are removed**	2^a^
	Donor	Neutral	The WT consensus sequence is not recognized	Neutral	The WT consensus sequence is not recognized		
c.1161+58A>G *MKKS/BBS6*	Acceptor	The WT consensus sequence is not recognized	Neutral	**Two new acceptor sites are created**	Neutral	Neutral	1
	Donor	Neutral	Neutral	**A new donor site is created**	Neutral		
c.11641C>T/p.(H3882Y) *ALMS1*	Acceptor	**A new acceptor site is created**	**One potential branch point is broken**	Neutral	Neutral	Neutral	**3**
	Donor	**Score for donor site increases from 0.92 to 0.93**		The WT consensus sequence is not recognized	**Score for donor site decreases from 1.3 to 1.2**		

Software abbreviations: *NNSplice (Neural Network SPLICE* 0.9 version from the Berkeley Drosophila Genome Project), *HSF (Human Splicing Finder 2.4.1 version)*,* ASSEDA (Automated Splice Site Exon Definition Server)* and *Rescue ESE (Relative Enhancer and Silencer Classification by Unanimous Enrichment)*. Any potential alteration on splicing process and ESE sequences were taken into account for score calculation (which is the total number of programs that yielded alterations). All the detected alterations are highlighted in bold. ^a^This intronic variant was not finally selected despite reaching a score of ‘2’, because only one splice site prediction tool (ASSEDA) gave positive results besides Rescue ESE prediction.

The missense variant p.(Y89C) in *BBS2* gene was identified in heterozygous state in the patient, who did not harbour any other change in the *BBS* genes analysed (data not shown). Its role in disease is unclear, as only Pmut software classified it as deleterious, but we consider several evidence of its pathogenicity exists: (i) it is absent in both 100 control chromosomes and the European ExAC database (http://exac.broadinstitute.org/), (ii) it segregates within the family (see Table 1), (iii) it is evolutionary conserved, and (iv) Tyr89 residue is located in a WD40 domain, known to be a site for protein–protein interactions (EMBL‐EBI database, http://www.ebi.ac.uk/). On the other hand, p.(H3882Y) in *ALMS1* gene was carried in heterozygous state by a clinically diagnosed BBS patient homozygote for the recurrent p.M390R mutation (*BBS1* gene). Although it is predicted to be benign at protein level, we consider its presumable effect on phenotype, as this patient showed typically ALMS features (dyslipidaemia and type 2 diabetes mellitus) not displayed by the non‐carrier sibling, also homozygote for p.M390R 15. In addition, the intronic change c.77‐6A>G in *BBS4* was selected as this family consists of two sibling homozygotes for p.(Q284*) mutation in *BBS10* gene 34, but only the girl is carrier of this presumably intronic polymorphism, which could have a modifier effect on phenotype. Finally, we included two nonsense mutations to check for nonsense‐mediated decay (NMD), especially suspected in the case of p.(G2*) mutation.

**Figure 1:**
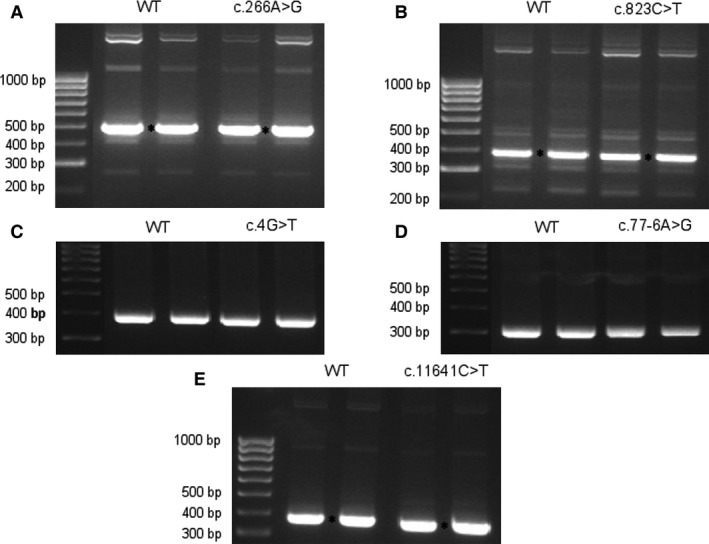
Results of minigene splicing assay for the five *BBS/ALMS1* putative splicing variants included. Agarose gel electrophoresis shows the band pattern of transcripts obtained after mRNA processing for each variant analysed, in duplicate. DNA marker sizes are indicated to the left of all pictures. Asterisks mark the canonical transcript when multiple bands were obtained (confirmed by DNA sequencing). All the variants produced unaltered splicing when comparing wild‐type and mutant. (**A**) c.266A>G/p.(Y89C) *BBS2* variant, (**B**) c.823C>T/p.(R275*) *BBS2* variant, (**C**) c.4G>T/p.(G2*) *ARL6/BBS3* variant, (**D**) c.77‐6A>G *BBS4* variant, (**E**) c.11641C>T/p.(H3882Y) *ALMS1* variant.

After performing minigene assay, we found that none of the five variants altered mRNA processing, as wild‐type and mutant constructions produced the same splicing pattern (Fig. 1). The canonical transcript was found and confirmed by direct sequencing in both wild‐type and mutant minigenes.

Interestingly, *BBS2* and *ALMS1* variants (wild‐type and mutant) generated several bands including the canonical transcript, especially noticed in *BBS2* gene. This would correspond to alternative splicing events using alternative sites not characterized and/or heteroduplex formations involving different isoforms. Unfortunately, we were not able to sequence the additional bands due to the weak signal.

On the other hand, minigene assay showed that NMD apparently did not work when nonsense variants were transcribed, as the canonical transcript with the corresponding point substitution was detected 36 hrs after transfection. This result suggests that these canonical transcripts containing premature termination codons (PTCs) would be translated into truncated proteins that negatively affect the normal cellular activity.

## Discussion

Until now, more than 500 mutations for BBS and 200 mutations for ALMS have been associated with these syndromes 7, 16. Moreover, the progressive implementation of next‐generation sequencing technologies in research and clinical practice is bringing to light thousands of variants which should be accurately classified through bioinformatics predictions and functional studies 12. All this information is contributing to make harder the challenging task of linking variants, phenotypes and clinical entities, which ultimately impairs proper genetic counselling and patient management in the already complex field of ciliopathies.

We have been working on mutational screening of BBS/ALMS patients for many years 24, 25, 34, 35. But now, we are interested in gaining knowledge about the biological role of all these changes and classify them properly, as there are few functional studies of variants related to BBS and ALMS that try to determine the effect of these mutations 36, 37, 38, 39. The recent data about the increasing proportion of splicing variants involved in genetic diseases 12, the absence of functional information for most *BBS*/*ALMS1* variants and the previous studies reporting minigene assay as a good approach to evaluate splicing alterations 12, 17, 40 have encouraged us to conduct this study.

After performing minigene assay, we did not find any difference regarding transcript processing between wild‐type and mutant constructions (Fig. 1) for variants c.77‐6A>G in *BBS4* and p.(H3882Y) in *ALMS1* gene, despite positive bioinformatics predictions (Table 2). Although triallelic inheritance had been proposed for these two families harbouring three segregating alleles, we cannot conclude that the distinct clinical presentation observed for the affected siblings is related to the third change found. Regarding p.(Y89C) variant, we cannot rule out the possibility of assigning a causal role to this variant if the second pathogenic mutation in *BBS2* gene was identified. Thus, a deep intronic mutation or a copy number variation could be present in the patient, but they have not yet been detected. Nor can we associate this change with splicing alterations, although we propose a potential modifier effect. This would be supported by the fact that it has been described mainly in heterozygous state, in South Asian populations only with an allele frequency of 0.0036 (ExAC database), suggesting a main role in modulating the phenotype.

The most interesting result corresponds to the nonsense mutations analysed here, p.(R275*) in *BBS2* and p.(G2*) in *ARL6/BBS3* gene, as degradation of PTC‐containing transcripts was not observed even when a PTC is introduced in the second codon (p.(G2*) mutation), in the absence of NMD inhibitors (Fig. 1). It has been described that transcripts containing an AUG‐proximal PTC can evade NMD and be translated into truncated proteins, mediated by the poly (A)‐binding protein 1, PABPC1, which can stabilize the mRNA and also have a repressive role 43; so our findings would support this model. It is possible, therefore, that these two nonsense mutations produce truncated BBS2 and ARL6/BBS3 proteins, whose function as important mediators in assembly and ciliary localization of BBSome, respectively, would be disrupted 42, 43. In addition, it is interesting to note that if p.(R275*) mutation produces a truncated BBS2, the protein functionality would be really compromised as the Arg275 residue is predicted to be located within a WD40 domain (EMBL‐EBI database, http://www.ebi.ac.uk/), known to be a site for protein–protein interactions involved in the assembly of protein complexes. This could be quite relevant, as BBS2 is a component of the BBSome core complex, an important intermediate in the assembly of the mature BBSome 43, essential for ciliogenesis and ciliary function.

However, our conclusions should be considered with caution, because minigene assay could differ from the real processing in affected tissues. Thus, cell‐ and tissue‐specific determinants could influence mRNA processing and NMD efficiency, such as isoforms balance, transcription rate or availability of splicing factors, what may be having a role in patients 44, 45 that is not reproducible under *in vitro* conditions. Moreover, other shortcomings should be taken into account, for example the difficulty of replicating the natural genomic context in genes such as *BBS* or *ALMS1*, typically presenting large introns and exons supposed to harbour important enhancer sites and other regulatory *cis‐*elements; refined approaches such as large minigenes systems could be more useful in this case. In addition, splicing prediction tools still show remarkable limitations to yield reliable predictions 21, 23, 48. Modelling *in vivo* using the zebrafish model, performing minigene assay in other specific cell lines such as RPE‐1 cells to check for tissue‐dependent differences or further molecular studies (e.g. RNA secondary structure analysis), could be useful for definitely elucidating the role of these variants, but cost‐effectiveness assessment should be considered.

The identification of new *BBS*/*ALMS1* splicing mutations, and maybe the reclassification of variants previously considered as pathogenic at protein level only from genomic sequence, is a worthwhile effort. This would be important not only for rare variants suspected to be disease‐causing, but also for common alleles classified as benign, which have been also described to interact with rare alleles to modulate the phenotype 36. Likewise, it would be interesting further to deepen in the identification of real splicing variants involved in BBS/ALMS with the perspective of developing new therapeutic approaches, poorly explored until now in relation to both syndromes (to our knowledge, there is only one study about splicing therapy for BBS using lentivirus, reported by Schmid *et al*., 47). Thus, an antisense oligonucleotides (AOs) strategy, which targets abnormal or cryptic splice sites and/or regulatory sequences trying to restore normal splicing, could represent a promising therapy as is mutation‐targeted and therefore suitable for private mutations 48, very frequent in BBS and ALMS, as has been reported for other diseases 48, 49, 50.

In conclusion, we report the first study of several *BBS* and *ALMS1* variants by minigene assay to clarify their biological role at splicing level. Functional studies of variants identified in BBS and ALMS patients are essential for their proper classification and subsequent genetic counselling and could also be the start point for new therapeutic approaches, currently based only on symptomatic treatment.

## Author contributions

This work was supported by a grant from *Fondo de Investigación Sanitaria del Instituto de Salud Carlos III‐*FEDER (PI12/01853). MAS (12/01442) and SCS (13/01835) received a graduate studentship award (*FPU fellowship*) from the Spanish Ministry of Education, Culture and Sports. MAS performed the bioinformatics predictions, minigene constructions and expression studies, analysed the data and wrote the manuscript. SCS participated in the minigene constructions and wrote the manuscript. GP participated in the bioinformatics study and wrote the manuscript. DV conceived of the study, participated in its design and coordination, and wrote the manuscript. All authors read and approved the final manuscript.

## Conflict of interest statement

The authors confirm that there are no conflict of interests.

## Supporting information


**Table S1** List of primers for amplification and cloning of inserts.

**Table S2** List of primers used for site‐directed mutagenesis.
