# Supplementary material for: Functional analysis by minigene assay of putative splicing variants found in Bardet–Biedl syndrome patients
Source: J Cell Mol Med. 2017 May 13;21(10):2268–75. doi: 10.1111/jcmm.13147 (PMC5618670; doi:10.1111/jcmm.13147)
Supplement: Supplementary file 1 — Table S1 List of primers for amplification and cloning of inserts. [file JCMM-21-2268-s001.doc]

| **VARIANT** | **GENE** | **EXON** | **FORWARD PRIMER (5´🡪3´)** | **REVERSE PRIMER (5´🡪3´)** | **AMPLICON SIZE (bp)** | **T_m_ (ºC)** |
| --- | --- | --- | --- | --- | --- | --- |
| c.266A>G/ p.(Y89C) | *BBS2* | 2 | AAGAATCTCGAGCCGTGATTTCATAATGACTG | AAGAATGCTAGCGCGATTAGCAACCATAACC | 613 | 56 |
| c.823C>T/ p.(R275*) | *BBS2* | 8 | AAGAATCTCGAGAGCTGGTTGAGGCTCTTTAG | AAGAATGCTAGCGCAAGTATAACCAGGTATAC | 525 | 58 |
| c.4G>T/ p.(G2*) | *ARL6/BBS3* | 2 | AAGAATCTCGAGGCTGCAAATCTGCGTGTCAG | AAGAATGCTAGCGCAAATTATGGCTGAGGATAC | 502 | 61 |
| c.77-6A>G | *BBS4* | 3 | AAGAATCTCGAGGAGAAACTGATATTACAAGC | AAGAATGCTAGCCTGATCTGAAAGCCCATGAG | 558 | 56 |
| c.11641C>T/ p.(H3882Y) | *ALMS1* | 17 | AAGAATCTCGAGAAGTCCTATCCACCTACAAC | AAGAATGCTAGCCAAACTTGCCATCAAACTTG | 481 | 57 |

**Table S1** List of primers for amplification and cloning of inserts

The resulting amplicon size and PCR annealing temperature are shown. Abbreviations: *bp*-base pairs. Tail for restriction sites recognition is highlighted in green. Enzyme restriction targets are shown in red (*CTCGAG* for *XhoI* and *GCTAGC* for *NheI*). Variant c.77-6A>G is located in intron 2-3 of *BBS4* gene. The following cDNA reference sequences were used: ENST00000245157 (*BBS2*), ENST00000463745 (*ARL6/BBS3*), ENST00000268057 (*BBS4*) and NM_015120.4 (*ALMS1*).
