# Supplementary material for: Functional analysis by minigene assay of putative splicing variants found in Bardet–Biedl syndrome patients
Source: J Cell Mol Med. 2017 May 13;21(10):2268–75. doi: 10.1111/jcmm.13147 (PMC5618670; doi:10.1111/jcmm.13147)
Supplement: Supplementary file 2 — Table S2 List of primers used for site‐directed mutagenesis. [file JCMM-21-2268-s002.doc]

**Table S2** List of primers used for site-directed mutagenesis

| **VARIANT** | **GENE** | **FORWARD PRIMER (5´🡪3´)** | **REVERSE PRIMER (5´🡪3´)** |
| --- | --- | --- | --- |
| c.266A>G/ p.(Y89C) | *BBS2* | TGAACCCTGAGCTTGGCT**G**TGATGCCCTTTTAGT | ACTAAAAGGGCATCA**C**AGCCAAGCTCAGGGTTCA |
| c.823C>T/ p.(R275*) | *BBS2* | GATGCTCGAAGTGAC**T**GAACTGGGGAGGTCA | TGACCTCCCCAGTTC**A**GTCACTTCGAGCATC |
| c.77-6A>G | *BBS4* | GTGTTGTTTGTTTTGTCAAAATATGCT**G**CCTAGCTCCAGAGTTT | AAACTCTGGAGCTAGG**C**AGCATATTTTGACAAAACAAACAACAC |
| c.11641C>T/ p.(H3882Y) | *ALMS1* | ACTTTTTGCAACAAGCAGAATGTA**T**ACATGTTAAACAAGGGCATAC | GTATGCCCTTGTTTAACATGT**A**TACATTCTGCTTGTTGCAAAAAGT |

Single nucleotide changes are highlighted in red bold. For c.4G>T/ p.(G2*) mutation, mutagenesis was not performed since we obtained both types of colonies, wild-type and mutant, after cloning. The following cDNA reference sequences were used: ENST00000245157 (*BBS2*), ENST00000463745 (*ARL6/BBS3*), ENST00000268057 (*BBS4*) and NM_015120.4 (*ALMS1*).
